# Single cell RNA sequencing reveals the role of local renin-angiotensin system in regulating ovarian physiological cycle and promoting PCOS

**DOI:** 10.1038/s41420-025-02531-8

**Published:** 2025-05-27

**Authors:** Lun Wei, Le Bo, Wangtao Jiang, Ruofan Qi, Chao Luo, Fei Qian, Panjie Ma, Jianping Qiu, Caiping Mao

**Affiliations:** 1 Reproductive Medicine Center, First Affiliated Hospital of Soochow University, Suzhou, Jiangsu China; 2 Department of Obstetrics and Gynaecology, The Affiliated Suzhou Municipal Hospital of Nanjing Medical University, Suzhou, Jiangsu China

**Keywords:** Endocrine reproductive disorders, Infertility

## Abstract

There is a local renin-angiotensin system (RAS) in the ovary, which is involved in regulating many important physiological processes, but the specific mechanism remains unclear. Polycystic ovarian syndrome (PCOS) is the most frequently reported non-iatrogenic condition with abnormal RAS expression, characterized by overweight or obesity and insulin resistance (IR), both of which are significantly correlated with many long-term complications. These conditions are closely linked to circulatory or local RAS, serving as potential common regulatory nodes. The present study analyzed single-cell RNA sequencing (scRNA-seq) data from mouse ovaries during the reproductive period to obtain the expression levels and location information of RAS components in all cell clusters. It further analyzed the cyclical fluctuations of RAS and the differential gene sets during the estrous cycle. Protein-protein interaction analysis predicted the most closely interacting pathway with RAS, and preliminary evidence of crosstalk between angiotensin II (AngII) and the insulin signaling pathway was identified in the scRNA-seq data. A PCOS mouse model was constructed, replicating clinical reproductive and metabolic complications, and the crosstalk between AngII and IRS1/PI3K/AKT was verified. In conclusion, this study revealed the dynamic changes of the ovarian local RAS at the cellular level during the estrous cycle, and described the role of RAS in regulating ovarian function from a single-cell perspective. It also provided evidence that IR, caused by the crosstalk between AngII and IRS1/PI3K/AKT pathways, may be a potential underlying mechanism of PCOS.

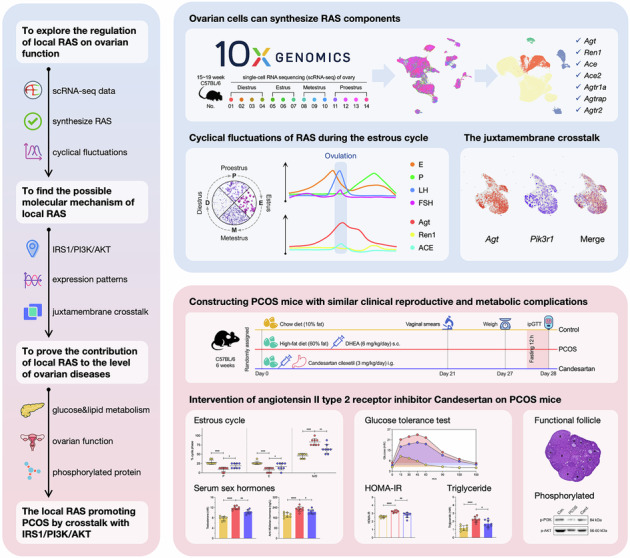

## Introduction

Originally, the renin-angiotensin system (RAS) was considered an endocrine system, consisting of substrates and enzymes from different tissues, which are widely involved in water-electrolyte metabolism and the cardiovascular system. It has gradually been found that relatively independent RAS exist in almost all organs or tissues, where they play important physiological roles through paracrine and autocrine mechanisms [1]. Up to now, the polypeptides, enzymes and receptors have been found to constitute two core pathways in RAS: the angiotensin II (AngII) and angiotensin 1-7 [Ang(1-7)] axes. These two pathways have opposing functions and together maintain the local microenvironment along with many bypass routes. Since the renal and genital tracts share common embryological origins, it is not surprising that cells from kidneys and gonads share similar molecular and functional properties [2]. As the largest extra-renal source of endogenous renin, the female reproductive system strongly suggests that the RAS plays a crucial role in regulating ovarian function [3]. It has been found that the ovarian RAS is involved in several key physiological processes, including determining the final fate of the follicle (growth to dominance and ovulation or atresia) [4–6], regulating ovarian steroidogenesis, and mediating the role of gonadotropins [7–9]. However, there is still no consensus on the exact mechanisms by which the RAS regulates ovarian function.

Abnormal expression and polymorphism of RAS components have been identified in several ovarian diseases, with polycystic ovarian syndrome (PCOS) being the most frequently reported non-iatrogenic condition [7, 8, 10–13]. PCOS is the most common endocrinopathy affecting reproductive-aged women, with a prevalence of 10 to 13% [14–17]. The etiology of PCOS is complex, with diverse clinical symptoms, including reproductive (oligoovulation or anovulation and polycystic ovaries), metabolic (overweight or obesity and hyperandrogenism), and psychological manifestations [18–22]. Overweight or obesity and insulin resistance (IR) are recognized as the key features of PCOS, which significantly correlate with many long-term complications, the most common of which are hypertension, type 2 diabetes, cardiovascular disease, and sleep disorders [14, 15, 18, 23]. Interestingly, these complications are closely related to the role of local RAS in other systems. At the same time, the 2023 PCOS guidelines emphasized the concept of “PCOS throughout the lifespan”, expanding management to include concern for the health of first-degree relatives, as evidence-based studies reveal a significantly increased risk of these diseases in this population [24, 25]. These multi-system associations and the genetic familial aggregation also confirm results from gene polymorphism analyses [10–13]. The common associated node among these conditions is probably the RAS, which has aroused our great interest and curiosity.

The single-cell RNA sequencing (scRNA-seq) is especially well-suited for investigating local signal pathways, such as ovarian RAS, which have been challenging to study previously [26–28]. Because collecting sufficient human ovarian tissue samples at childbearing age for each phase of the estrous cycle is difficult, studies often turn to animal models. Since the physiological changes in the ovaries during the estrous cycle are similar between humans and other mammals, Ivana and colleagues were the first to complete scRNA-seq on C57BL/6 mice at each estrous cycle phase [29]. Our research was built on this work, analyzing the expression of RAS at the cellular level for the first time. The present study was devised to explore the regulation of local RAS on ovarian function and its possible molecular mechanism. More importantly, our evidence not only helps to understand the pathological process of PCOS, but also provides groundbreaking proof of the contribution of RAS to ovarian diseases.

## Results

### Expression of RAS component in ovarian cells

**Fig. 1:**
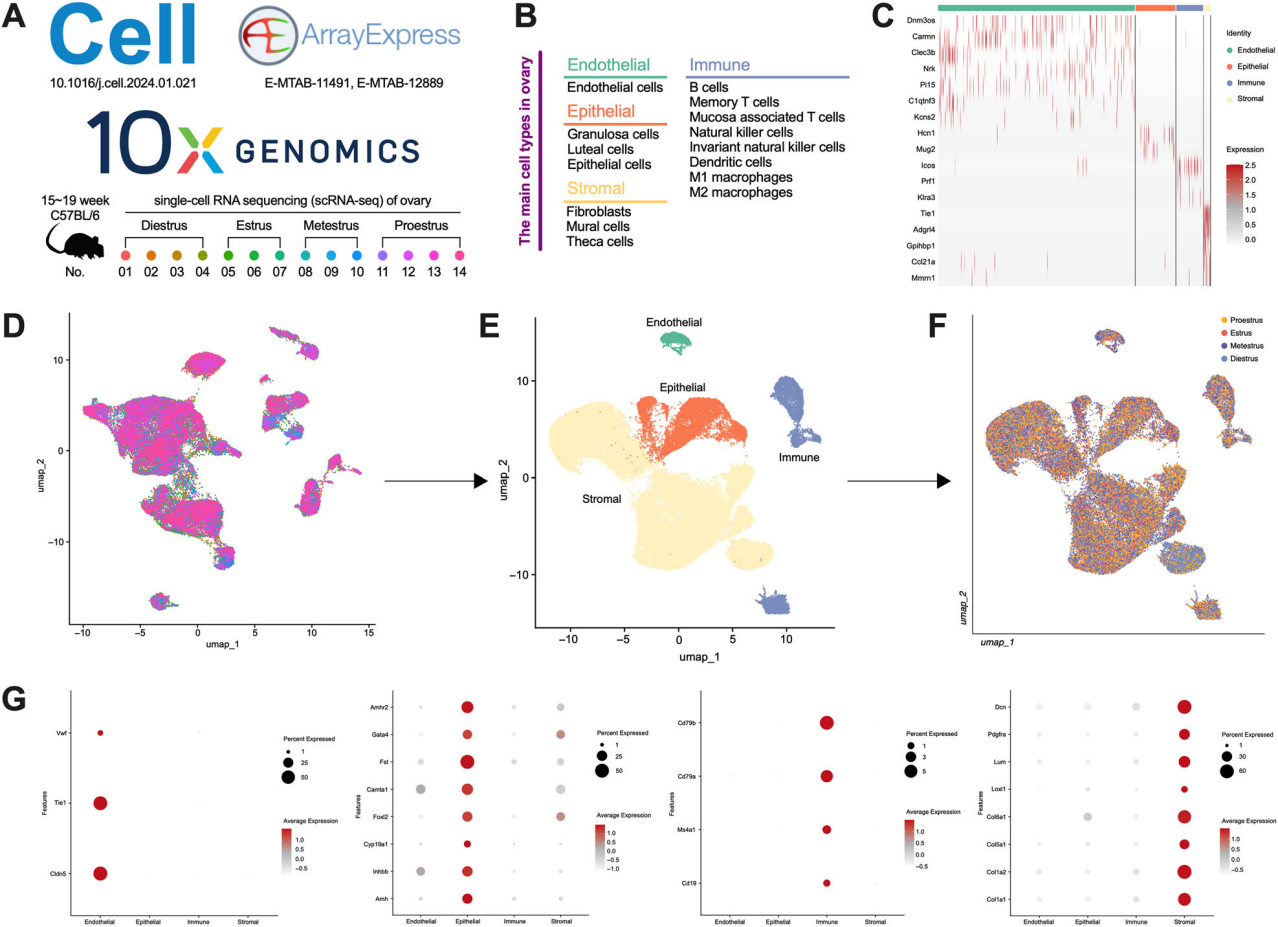
The pretreatment and cell clustering of single-cell RNA sequencing (scRNA-seq) data. **A** Methodological and biological characteristics of the scRNA-seq data. **B** Detailed description of cell and subcellular clustering. **C** Differentially expressed genes heatmap of cell clustering. Umap of the scRNA-seq data after preprocessing (**D**) and cell clustering (**E**). **F** Umap of the scRNA-seq data at each phase of the estrous cycle. **G** Detection of major cell marker genes in each cluster (endothelial cells, granulosa cells, B cells, fibroblasts in turn).

The data of scRNA-seq were collected from 14 female C57BL/6 mice aged 15–19 weeks. The mice were categorized into four phases of the estrous cycle through vaginal smears, comprising 4 in diestrus (D), 3 in estrus (E), 3 in metestrus (M) and 4 in proestrus (P) (Fig. 1A). The ovarian cells were classified into four main categories, as defined by the data source [29]: epithelium, stroma, endothelium and immune categories (Fig. 1B). After preprocessing, the cells were clustered according to these categories, and all cell clusters were tested (Fig. 1C). The initial UMAP visualization showed the distribution of cells, distinguished by individual mice (Fig. 1D). These cells were then further classified by cell type after clustering (Fig. 1E), and according to each phase of the estrous cycle (Fig. 1F). Finally, the clustering quality was confirmed through the expression of key marker genes specific to the primary cell types, such as endothelial cells (*Cldn5, Tie1, Vwf*), granulosa cells (*Amh, Inhbb, Cyp19a1*), B cells (*Cd19, Ms4a1, Cd79a, Cd79b*), and fibroblasts (*Col1a1, Col1a2, Col5a1*) (Fig. 1G).

**Fig. 2:**
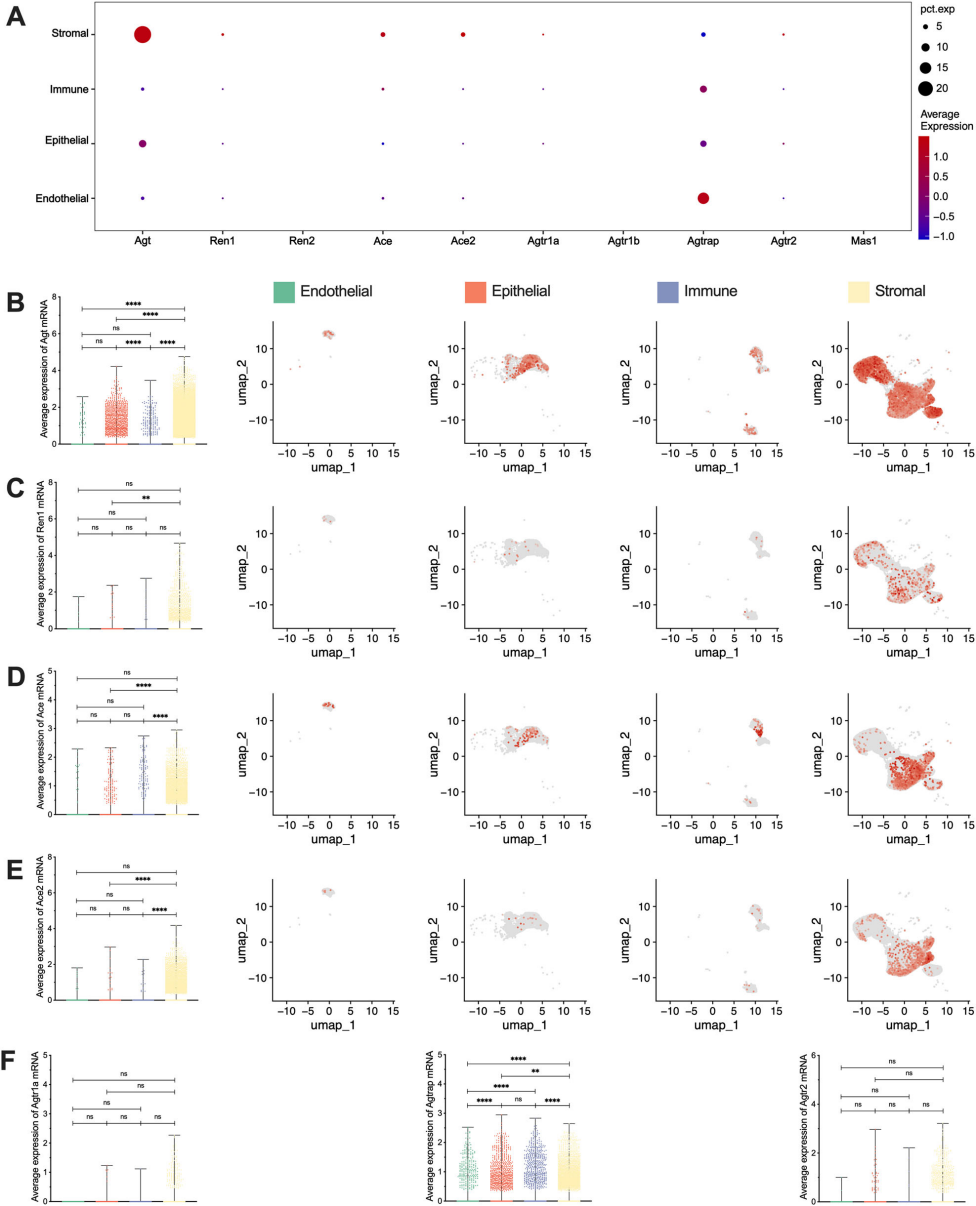
Expression of RAS (renin-angiotensin system) main components and receptors in each cell cluster. **A** Overall bubble chart of RAS main components and receptors in each cluster. **B**–**E** Differentially expressed Agt, Ren1, Ace, and Ace2 mRNA and its location. From left to right: the visual statistical results and the expression positions of the 4 parts. **F** Differentially expressed Agtr1a, Agtrap, and Agtr2 mRNA. **p < 0.01, ****p < 0.0001, “ns” for non-significant results.

Local RAS of ovary is a large, complex, precise, and dynamic system. In simple terms, renin hydrolyzes angiotensinogen (AGT) to produce angiotensin I (AngI), and then AngII and Ang(1-7) are produced through angiotensin converting enzyme (ACE) and ACE2, and finally act on their respective receptors. Quantitative and spatial analysis of RAS components in the clustered data revealed that RAS is extensively expressed in ovarian cells (Fig. 2A). Specifically, *Agt* (angiotensinogen, *P* < 0.0001), *Ren* (renin, *P* = 0.0025), *Ace* (angiotensin-converting enzyme, *P* < 0.0001) and *Ace2* (angiotensin-converting enzyme 2, *P* < 0.0001) exhibited highly expressed levels in stromal cells, with *Agt* also showing high expression in epithelial cells (*P* < 0.0001) (Fig. 2B–E). At the same time, a certain degree of spatial clustering was observed in cells expressing RAS components, particularly in stromal and epithelial cells. This suggests the existence of sub-clustered cells or perhaps cells at specific physiological stages. Additionally, RAS receptors were expressed in ovarian cells, but there was no significant difference between cell clusters (*P* > 0.05) (Fig. 2F). Notably, *Agtrap* (AngII type 1 receptor-associated protein) was most highly expressed in endothelial cells (*P* < 0.0001), followed by immune (*P* < 0.0001) and epithelial cells (*P* = 0.0060), and lowest in stromal cells. These data suggested that RAS components are widely expressed in ovarian cells, which have the potential to form local RAS independent of circulation. Furthermore, stromal and epithelial cells need more attention, which are the main functional cells, further exploring the relationship between RAS and ovarian function during the estrous cycle.

### RAS in stromal & epithelial sub-clusters and cyclical fluctuations during the estrous cycle

**Fig. 3:**
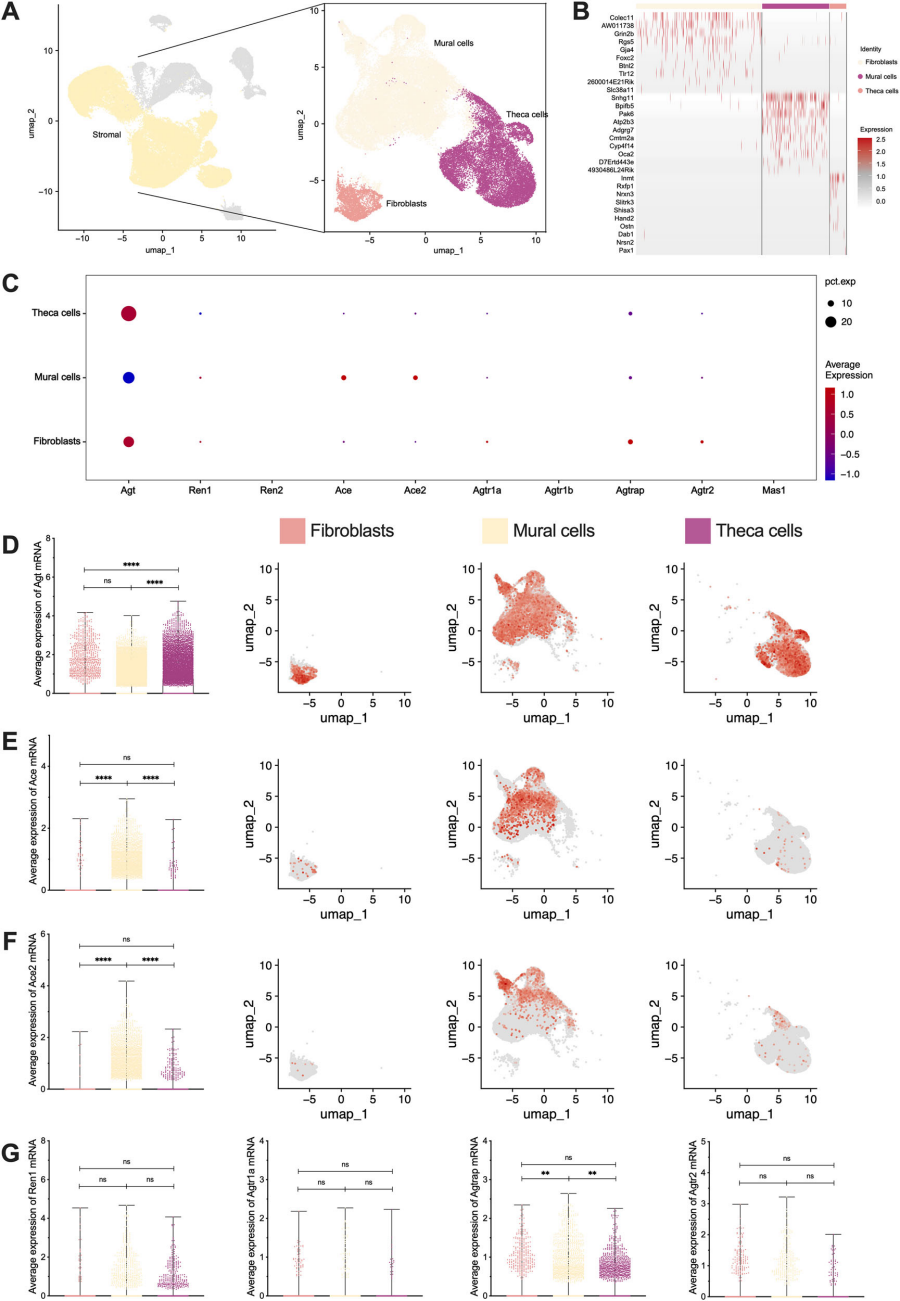
Subcellular clustering of stromal and expression of RAS main components and receptors. **A** Subcellular clustering from stromal, including fibroblasts, mural, and theca cells. **B** Differentially expressed genes heatmap of subcellular clustering in stromal. **C** Overall bubble chart of RAS main components and receptors in each sub-cluster. **D**–**F** Differentially expressed Agt, Ace, and Ace2 mRNA and its location. From left to right: the visual statistical results and the expression positions of the 3 parts. **G** Differentially expressed Ren1, Agtr1a, Agtrap, and Agtr2 mRNA. **p < 0.01, ****p < 0.0001, “ns” for non-significant results.

Further clustering of stromal cells revealed subtypes, including fibroblasts, mural cells, and theca cells (Fig. 3A), and these sub-clustered cells were tested (Fig. 3B). Quantitative analysis revealed widespread expression of RAS components in stromal sub-cluster cells (Fig. 3C). Specifically, *Agt* was most highly expressed in theca cells (*P* < 0.0001) (Fig. 3D), while *Ace* (*P* < 0.0001) and *Ace2* (*P* < 0.0001) expression was significantly higher in mural cells (Fig. 3E, F). There was no significant difference in *Renin* and RAS receptor expression among these sub-clusters (*P* > 0.05) (Fig. 3G). Additionally, the expression of *Agtrap* was highest in fibroblasts (*P* = 0.0043), followed by theca cells (*P* = 0.0089), and the lowest in mural cells.

**Fig. 4:**
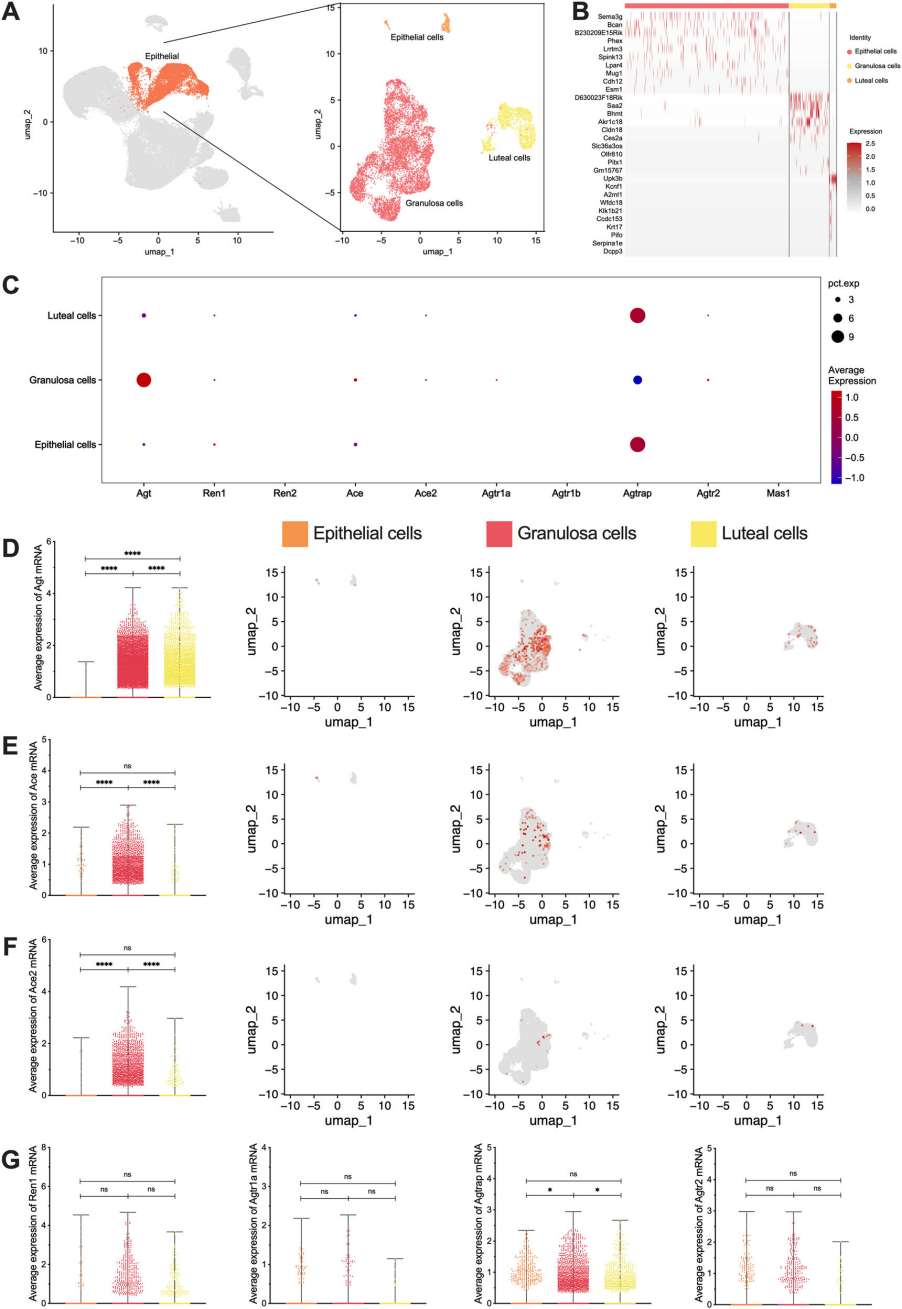
Subcellular clustering of epithelial and expression of RAS main components and receptors. **A** Subcellular clustering from epithelial, including epithelial, granulosa and luteal cells. **B** Differentially expressed genes heatmap of subcellular clustering in epithelial. **C** Overall bubble chart of RAS main components and receptors in each sub-cluster. **D**–**F** Differentially expressed Agt, Ace, and Ace2 mRNA and its location. From left to right: the visual statistical results and the expression positions of the 3 parts. **G** Differentially expressed Ren1, Agtr1a, Agtrap, and Agtr2 mRNA. *p < 0.05, ****p < 0.0001, “ns” for non-significant results.

Similarly, epithelial cells were further clustered into epithelial cells, granulosa cells, and luteal cells (Fig. 4A), and these sub-clustered cells were tested (Fig. 4B). Quantitative analysis of RAS components in epithelial sub-cluster cells also showed widespread expression (Fig. 4C). Specifically, *Agt* expression was found to be the highest levels in granulosa and luteal cells (*P* < 0.0001) (Fig. 4D), while *Ace* (*P* < 0.0001) and *Ace2* (*P* < 0.0001) were significantly higher in granulosa cells (Fig. 4E, F). There was no significant difference in *Renin* and RAS receptor expression in these sub-clusters (*P* > 0.05) (Fig. 4G). In addition, the expression of *Agtrap* was highest in epithelial cells (*P* = 0.0263), followed by luteal cells (*P* = 0.0130), and lowest in granulosa cells.

**Fig. 5:**
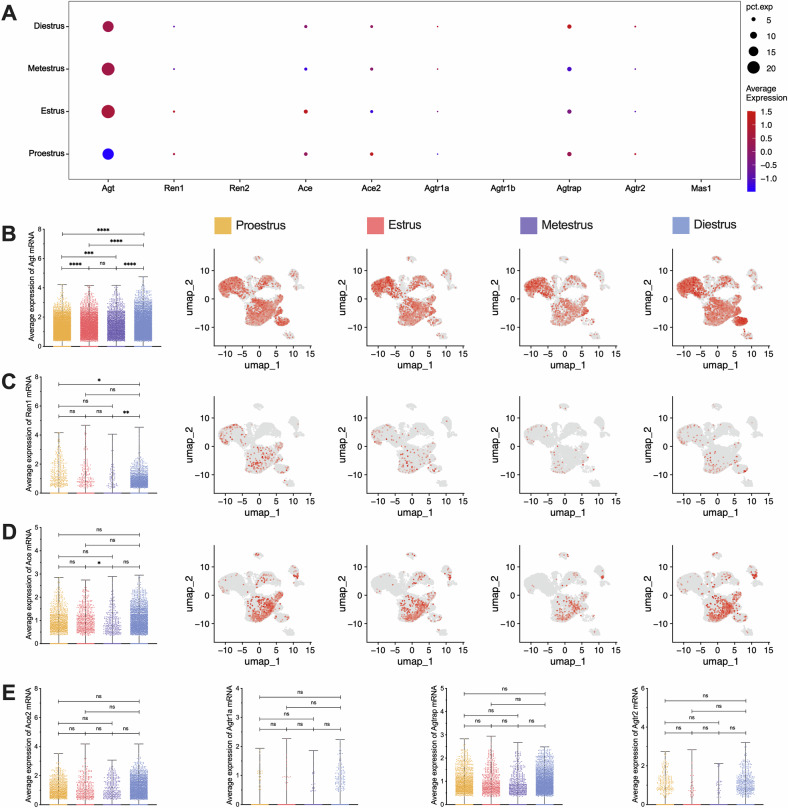
Expression of RAS main components and receptors at each phase of the estrous cycle. **A** Overall bubble chart of RAS main components and receptors at each phase of the estrous cycle. **B**–**D** Differentially expressed Agt, Ren1, and Ace mRNA and its location in all clusters. From left to right: the visual statistical results and the expression positions of the 4 parts. **E** Differentially expressed Ace2, Agtr1a, Agtrap, and Agtr2 mRNA. *p < 0.05, **p < 0.01, ***p < 0.001, ****p < 0.0001, “ns” for non-significant results.

Beyond the localization and characterization of sub-clustered cells, we also analyzed the cyclical expression patterns of RAS components throughout the estrous cycle (Fig. 5A). The expression of *Agt* showed significant cyclical variation, with the highest levels observed in E (*P* < 0.0001), followed by a decrease in M (*P* = 0.0005), reaching the lowest level in D (*P* < 0.0001). *Agt* levels gradually increased in P, peaking again in the next estrus cycle (Fig. 5B). At the same time, the expression of *Renin* peaked in D (*P* = 0.0047) (Fig. 5C), while *Ace* reached its highest level in E (*P* = 0.0110) (Fig. 5D). Moreover, no significant cyclical changes were observed in *Ace2* or RAS receptor expression (*P* > 0.05) (Fig. 5E). The core components of RAS are highly expressed in ovarian functional cells, exhibiting distinct cyclical fluctuations throughout the estrous cycle. The evidence strongly indicates that the local RAS functions as a key regulator in the physiological functioning of the ovary.

### Preliminary evidence of crosstalk between ovarian RAS and IRS1/PI3K/AKT signaling pathways

**Fig. 6:**
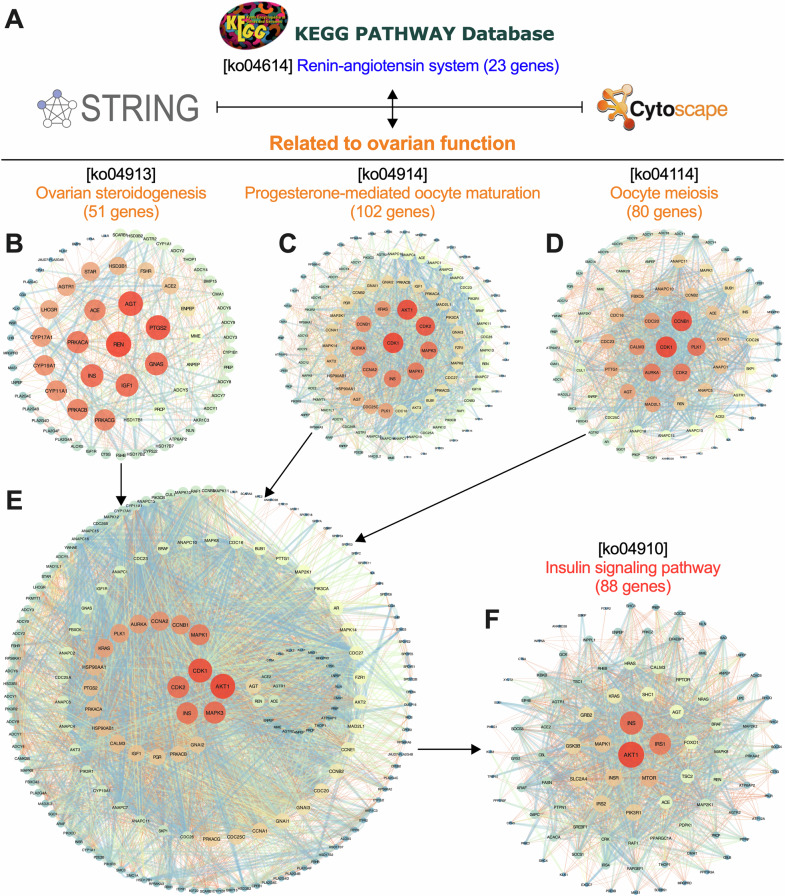
Protein-protein interaction (PPI) analysis of RAS and insulin signaling pathway based on the above GSEA results. **A** Selecting gene sets from KEGG PATHWAY Database for PPI analysis. PPI analysis of gene sets related to RAS and ovarian steroidogenesis (**B**), progesterone-mediated oocyte maturation (**C**), oocyte meiosis (**D**), all ovarian function (**E**), insulin signaling pathway (**F**), respectively. Each node represents a protein, and the size and color of the node represent the Degree of protein (the color changes from red to blue as the number of interactions with other protein decreases).

Initially, we endeavored to elucidate the potential mechanism through which RAS modulates ovarian physiological processes via the analysis of the protein-protein interaction (PPI) networks (Fig. 6A). Specifically, the RAS gene set from the KEGG PATHWAY Database, designated as [ko04614], was chosen for analysis of its interactions with related ovarian functional pathways. These pathways include Ovarian Steroidogenesis [ko04913], Progesterone-Mediated Oocyte Maturation [ko04914], and Oocyte Meiosis [ko04114] (Fig. 6B–D). Subsequently, we conducted a comprehensive analysis of all gene sets associated with ovarian functional pathways. The results showed that hub genes integral to ovarian function, including *AKT1, CDK1, CDK2, MAPK3*, and *INS*, exhibited close interactions with RAS-related genes such as *AGT, REN, ACE, ACE2,* and *AGTR1* (Fig. 6E). This revealed that local RAS is closely linked to the ovarian insulin pathway. Finally, we further analyzed the interaction between Insulin Signaling Pathway [Ko04910] and RAS, revealing that hub genes, including *AKT1, INS, IRS1, MTOR, INSR,* and *MAPK1*, play a crucial role in this interaction (Fig. 6F).

**Fig. 7:**
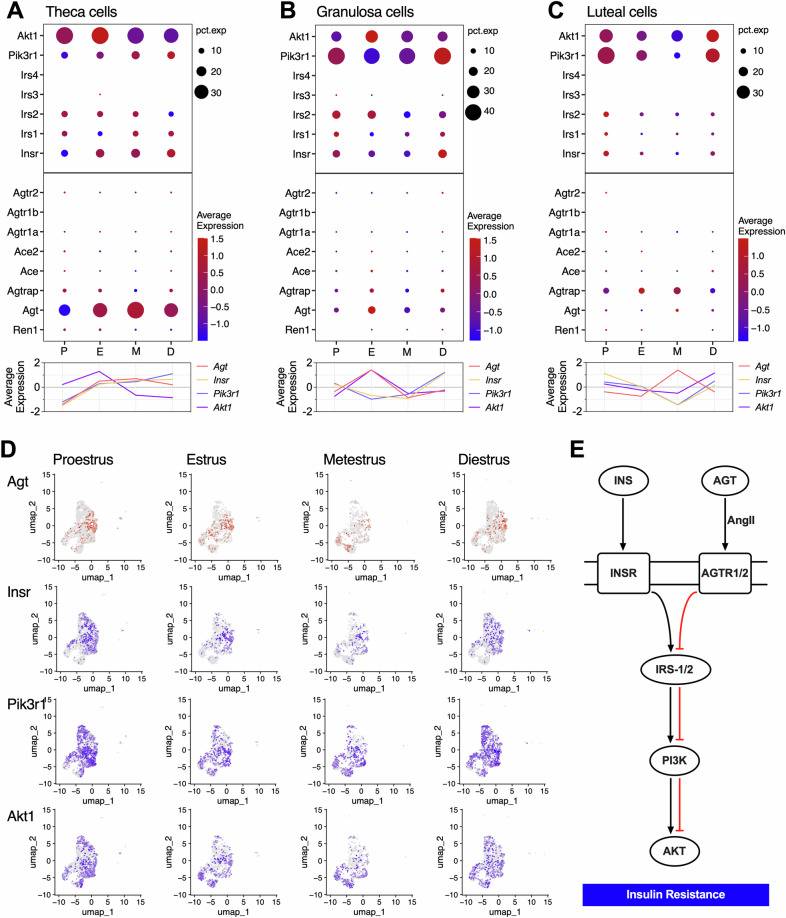
Preliminary evidence of crosstalk between ovarian local RAS and IRS1/PI3K/AKT in insulin signaling pathway. **A**–**C** Overall bubble chart of RAS main components and IRS/PI3K/Akt signaling pathway in theca, granulosa, and luteal cells. **D** Localization of Agt, Irs1, Pik3r1, and Akt1 mRNA expression in theca cells. **E** The hypothesis of crosstalk between ovarian local RAS and IRS/PI3K/Akt signaling pathway.

Based on these findings, we further analyzed the expression patterns of the ovarian RAS and insulin pathways, focusing on their cyclical fluctuations and cellular localization. It was found that the expression of *Insr*, *Pik3r1* and *Akt1* were closely associated with *Agt* (Fig. 7A, B), but displayed divergent trajectories in luteal cells at the same phase, particularly *Pik3r1* (Fig. 7C). Subsequently, using theca cells as an example, we observed that cells expressing both RAS and insulin pathways components were highly clustered (Fig. 7D), providing evidence for crosstalk between these pathways at the juxtamembrane level. It’s known that crosstalk between RAS and insulin pathways has been confirmed in non-ovarian cells [30–34]. Specifically, AngII activates its receptor AT1R (AngII type1 receptor), which inhibits insulin pathway activation and ultimately leads to IR [30, 35] (Fig. 7E). Based on our findings, we hypothesize that a similar crosstalk between ovarian RAS and insulin pathways may occur. These data provided preliminary evidence of a close relationship between RAS and the insulin pathway in the ovary, establishing a molecular basis for the crosstalk between ACE/AngII/AT1R and IRS1/PI3K/AKT at the juxtamembrane level.

### Constructing and treating PCOS mice with similar clinical reproductive and metabolic complications

**Fig. 8:**
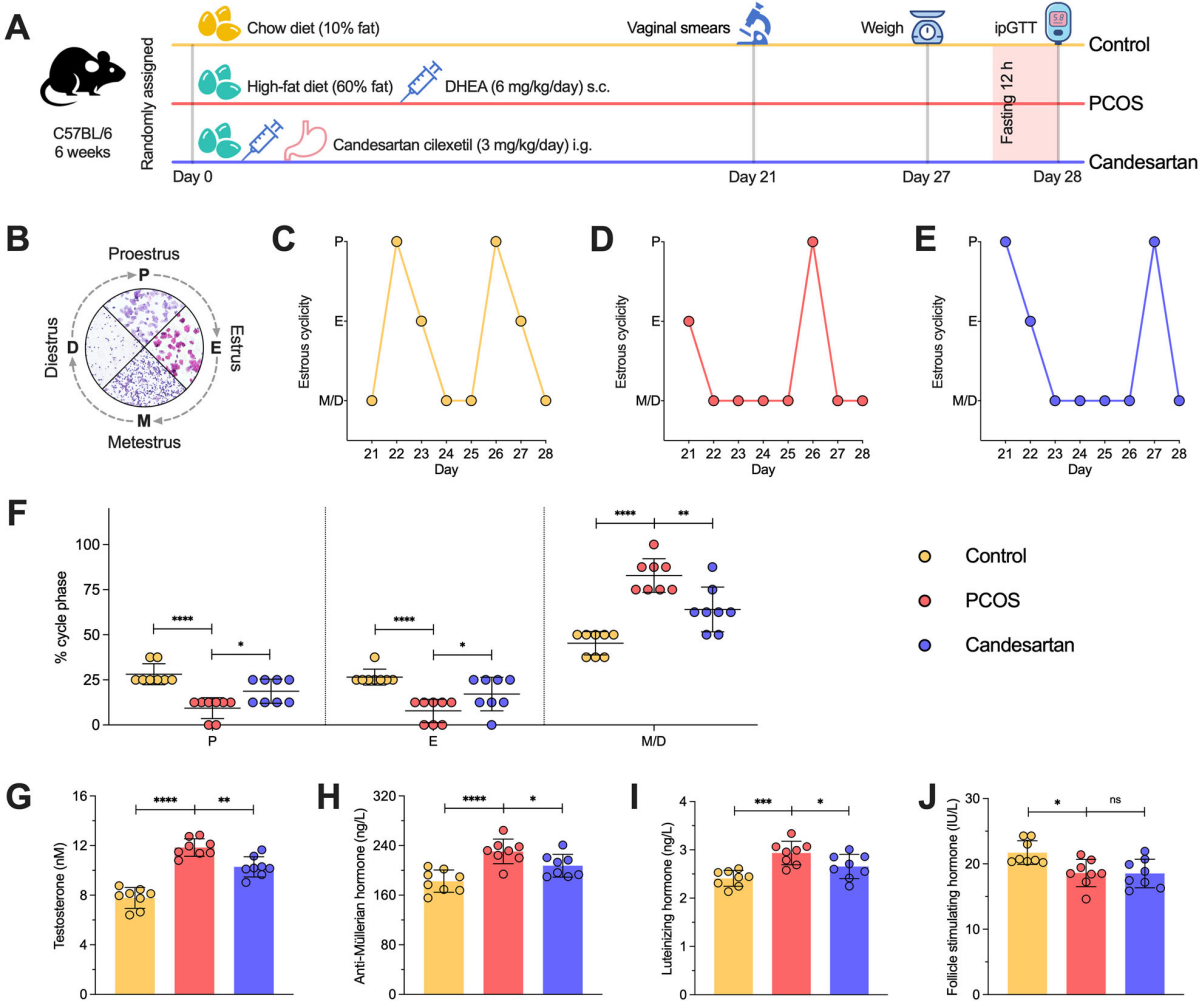
Constructing polycystic ovarian syndrome (PCOS) mouse model and monitoring the estrous cycle, serum sex hormone level. **A** The protocol of constructing and testing PCOS mouse model. **B** Representative microscopic images of the complete estrous cycle. **C**–**E** Representative estrous cyclicity of mice in the Control, PCOS, and Candesartan group during 8 consecutive days. **F** Proportion of each stage during 8 consecutive days. **G**–**J** Serum testosterone, anti-Müllerian hormone, luteinizing hormone and follicle stimulating hormone levels in mice. *n* = 8 per group, each point represents a sample, Control and Candesartan both vs. PCOS, *p < 0.05, **p < 0.01, ***p < 0.001, ****p < 0.0001, “ns” for non-significant results.

One of the most prevalent murine models for PCOS is induced using Dehydroepiandrosterone (DHEA), which leads to the development of characteristics similar to human PCOS, such as polycystic ovaries, anovulation and hyperandrogenism [36]. Moreover, high-fat diet (HFD)-treated mice have also been used as a PCOS model, exhibiting infertility [37, 38]. Consequently, as previously reported [39], the co-administration of DHEA and HFD can induce reproductive and metabolic disturbances that mimic the clinical features of PCOS [40] (Fig. 8A). Starting on day 21 after completing the modeling, the estrous cycle of the mice was monitored daily via vaginal smear. Representative images of each phase of the estrous cycle were shown in Fig. 8B. The estrous cycle of the Control group was characterized by four regular phases (Fig. 8C), whereas the PCOS and Candesartan (AT1R inhibitor) groups showed disordered cycles (Fig. 8D, E). Further calculation of the proportion of each phase showed that, compared with the Control group, the P and E phases significantly decreased in the PCOS group (*P* < 0.0001), while the M/D phases increased (*P* < 0.0001). After Cand. treatment, the proportion of P and E phases increased (*P* = *0.0108; P* = *0.0265*), and the M/D phases decreased (*P* = 0.0017) (Fig. 8F). Concurrently, serum sex hormone analyses revealed that the levels of testosterone (T, *P* < 0.0001), anti-Müllerian hormone (AMH, *P* < 0.0001), and luteinizing hormone (LH, *P* = 0.0002) were markedly elevated in the PCOS group compared to the Control group (*P* < 0.01), but decreased after Cand. intervention (*P* = 0.0013*; P* = 0.0423*; P* = 0.0379) (Fig. 8G–I). In addition, changes of follicle-stimulating hormone (FSH) were observed only in the PCOS group (*P* = 0.0112), with no significant effect from Cand. (*P* = 0.9963) (Fig. 8J).

**Fig. 9:**
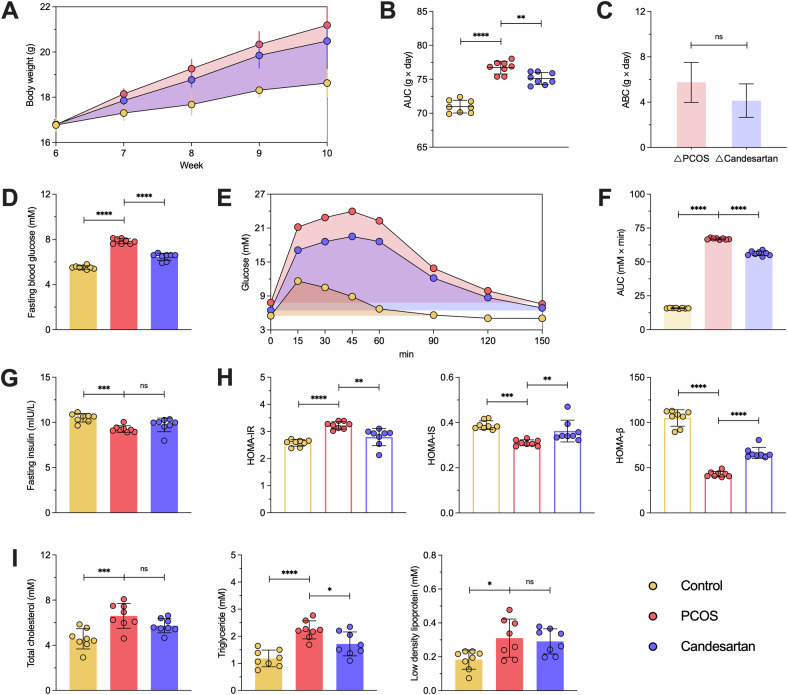
The trajectory of body weight and the level of glucose-lipid metabolism in mice. **A**–**C** The trajectory of body weight in mice, and statistical analysis of the area under the curve (AUC) and the area between the curves (ABC). **D**, **G**–**H** Serum fasting blood glucose and insulin levels in mice, and HOMA-IR, HOMA-IS and HOMA-β were calculated. **E**, **F** The trajectory of glucose tolerance test by intraperitoneal injection (ipGTT) and statistical analysis of the area under the curve AUC. **I** Serum total cholesterol, triglyceride and low density lipoprotein levels in mice. *n* = 8 per group, each point represents a sample, Control and Candesartan both vs. PCOS, ^ns^*p* > 0.05, *p < 0.05, **p < 0.01, ***p < 0.001, ****p < 0.0001, “ns” for non-significant results.

Subsequently, we also tested a series of metabolic indicators. With dietary intervention, the body weight gap between the Control and PCOS groups gradually widened (Fig. 9A). By calculating the area the under curve (AUC), it was found that the PCOS group showed a much larger increase in body weight compared to the Control group (*P* < 0.0001), and Cand. had a significant effect on body weight control (*P* = 0.0040) (Fig. 9B). However, further analysis of body weight showed no reduction in HFD-induced weight gain after Cand. treatment (*P* = 0.0667) (Fig. 9C). After 12 h of fasting, the fasting blood glucose (FBG) in the PCOS group was significantly higher than that in the Control group (*P* < 0.0001), and Cand. intervention effectively improved FBG levels (*P* < 0.0001) (Fig. 9D). Following an intraperitoneal glucose injection, blood glucose levels were monitored continuously (Fig. 9E). Similarly, the calculated AUC indicated significantly higher glucose levels in the PCOS group compared to the Control group (*P* < 0.0001), with a marked improvement after Cand. treatment (*P* < 0.0001) (Fig. 9F). Finally, fasting insulin (FINS) levels were also significantly higher in the PCOS group compared to the Control (*P* = 0.0004), but Cand. intervention had no significant effect (*P* = 0.1877) (Fig. 9G).

Homeostatic model assessment (HOMA) is a method for assessing beta-cell function and IR based on fasting glucose and insulin levels. Currently, the HOMA model has become a widely used tool in clinical, experimental, and epidemiological studies. Further calculations showed that HOMA-IR (*P* < 0.0001), HOMA-IS (*P* < 0.0001), and HOMA-β (*P* < 0.0001) indices changed significantly in the PCOS group and showed significant improvement with Cand. treatment (*P* = 0.0010; *P* = 0.0056; *P* < 0.0001) (Fig. 9H). In addition, blood lipid levels, including total cholesterol (TC, *P* = 0.0004), triglyceride (TG, *P* < 0.0001), and low-density lipoprotein (LDL, *P* = 0.0127), were significantly higher than the Control group, though Cand. intervention only modestly improved TG levels (*P* = 0.0184) (Fig. 9I). These data suggested that we have successfully constructed PCOS mice with similar clinical reproductive and metabolic complications. Furthermore, blocking the AngII receptor can ameliorate the reproductive and metabolic complications in PCOS mice.

### The crosstalk between ovarian RAS and IRS1/PI3K/AKT pathways in the PCOS process

**Fig. 10:**
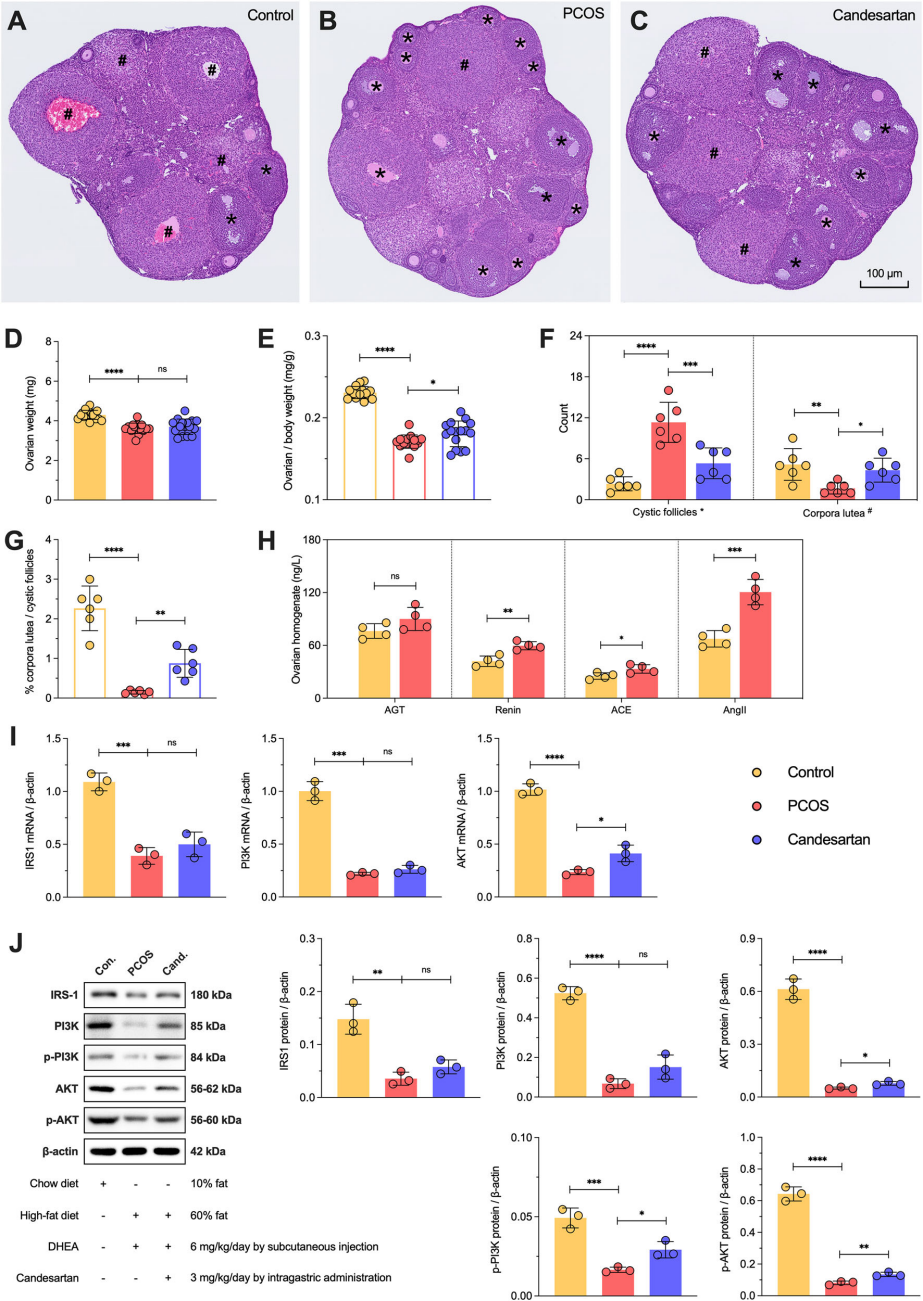
Histology and morphology of mouse ovary, and the expression level of IRS1/PI3K/AKT signaling pathway. **A**–**C** Representative images of the Control, PCOS, and Candesartan group ovarian tissue sections stained by H&E (*n* = 6 per group). * show cystic follicles and ^#^ corpora lutea in microscopic images. **D**, **E** Single ovarian weight and the ratio of ovarian to body weight (*n* = 16 per group). **F**, **G** The counting and proportion of cystic follicles and corpora lutea (*n* = 6 per group). **H** The transcription and translation level of IRS1/PI3K/AKT mRNAs and proteins in mouse ovary (*n* = 4 per group). **I** The transcription level of IRS1/PI3K/AKT mRNAs in mouse ovary (*n* = 3 per group). **J** Representative images of western blot and the translation level of IRS1/PI3K/AKT (phosphorylated) proteins in mouse ovary (*n* = 3 per group). Each point represents a sample, Control and Candesartan both vs. PCOS, *p < 0.05, **p < 0.01, ***p < 0.001, ****p < 0.0001, “ns” for non-significant results.

In this part, we performed a more in-depth analysis of the ovary. First of all, we examined the ovarian tissue sections from the three groups using histomorphology, with representative images shown in Fig. 10A–C. Overall, the ovarian weight in the Control group was significantly higher than that in the PCOS group (*P* < 0.0001), but there was no statistical difference between the PCOS and Cand. groups (*P* = 0.7340) (Fig. 10D). Further calculation of the ovarian organ index indicated that the value in the Control group remained significantly higher than that in the PCOS group (*P* < 0.0001), and this ratio was partially restored after Cand. treatment (*P* = 0.0363) (Fig. 10E). Then, the number of cystic follicles and corpora lutea were also counted. In short, cystic follicles are typical clinical manifestations (polycystic ovarian morphology), while corpora lutea result from functional ovulation. Compared with the Control group, the PCOS group had significantly more cystic follicles (*P* < 0.0001) and fewer corpora lutea (*P* = 0.0063). After Cand. intervention, the number of cystic follicles (*P* = 0.0006) and corpora lutea (*P* = 0.0331) recovered compared to the PCOS group (Fig. 10F). Further analysis of the ratio between corpora lutea and cystic follicles suggested impaired follicular development in the PCOS group compared to the Control group (*P* < 0.0001), with partial improvement following Cand. treatment (*P* = 0.0089) (Fig. 10G).

Finally, we examined the predicted crosstalk mechanism at the molecular level. In ovarian homogenate, the renin (*P* = 0.0034), ACE (*P* = 0.0382), and AngII (*P* = 0.0008) levels in the PCOS group were significantly higher than in the Control group, although AGT levels showed no statistical difference (*P* = 0.1291) (Fig. 10H). Subsequently, from the transcriptional level, ovarian *IRS1* (*P* = 0.0005)*, PI3K* (*P* = 0.0001), and *AKT* (*P* < 0.0001) mRNA expression in the PCOS group was significantly reduced, while Cand. treatment only led to a slight improvement in *AKT* (*P* = 0.0204) (Fig. 10I). Fortunately, at the protein level, more detailed and direct evidence was obtained; changes in IRS1 (*P* = 0.0032), PI3K (*P* < 0.0001) and AKT (*P* < 0.0001) protein levels were consistent with mRNA transcription, with significantly lower levels in the PCOS group compared to the Control group. Additionally, phosphorylated PI3K (*P* = 0.0010) and AKT (*P* < 0.0001) protein levels were significantly lower in the PCOS group compared to the Control group (Fig. 10J). Although IRS1 (*P* = 0.1001) and PI3K (*P* = 0.0942) protein levels showed no statistical difference after Cand. intervention, AKT (*P* = 0.0165) and phosphorylated PI3K (*P* = 0.0156) & AKT (*P* = 0.0063) protein levels increased significantly. These data strongly suggested that the AngII axis is overactivated and promoting PCOS through crosstalk with the IRS1/PI3K/AKT signaling pathway.

## Discussion

**Fig. 11:**
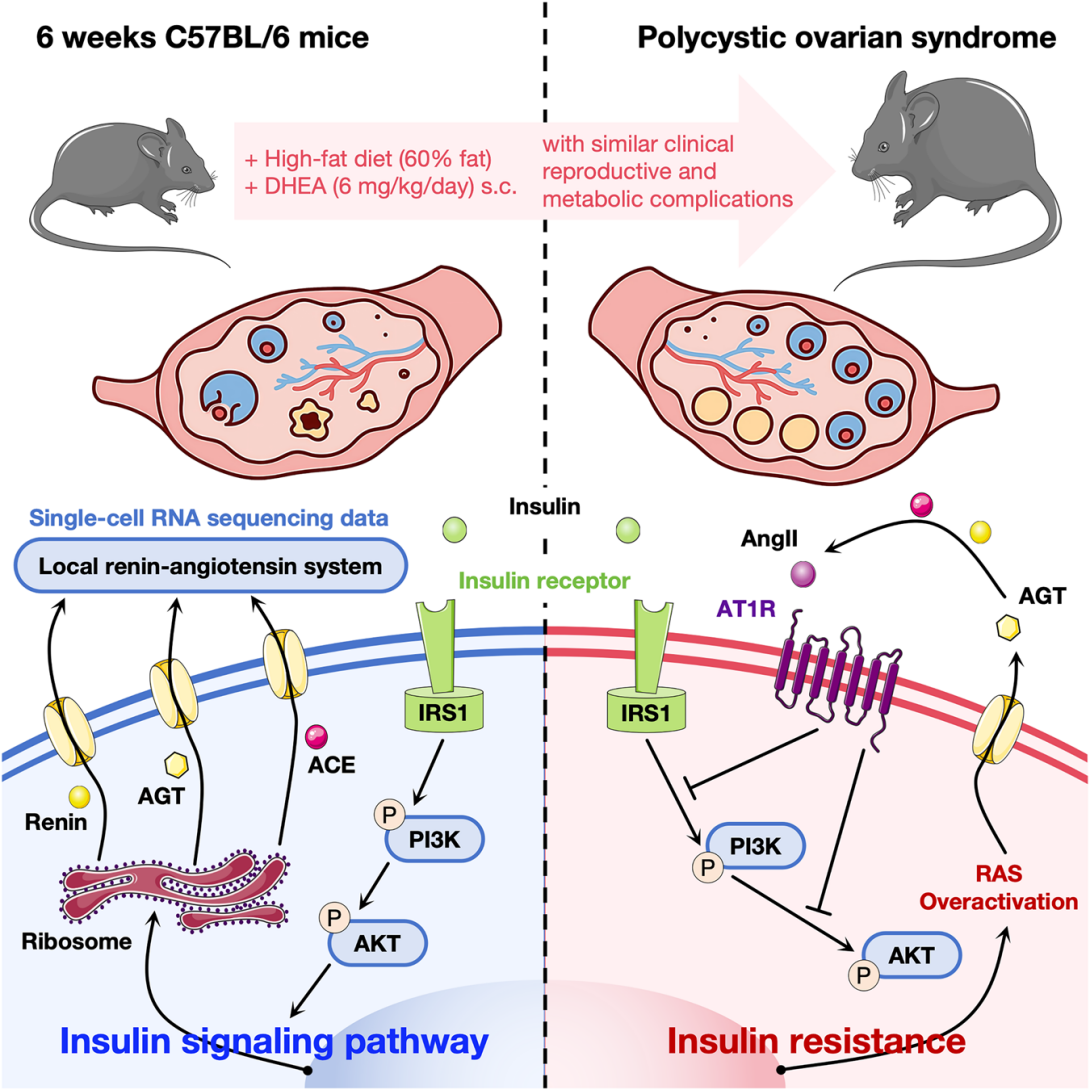
Ovarian RAS promoting PCOS by crosstalk with the IRS1/PI3K/AKT.

In this study, we conducted the first systematic observation of the cellular-level expression and distribution of ovarian RAS, utilizing single-cell RNA sequencing data from mouse ovaries during the reproductive phase. Cyclical changes in RAS components throughout the estrus cycle were demonstrably found. From the perspective of downstream protein function, we tried to explain the ovarian physiological process in which RAS may be involved and found preliminary evidence of crosstalk between local RAS and the insulin signaling pathway. We constructed a mouse model of PCOS, which demonstrated that the overactivated AngII axis in ovarian RAS interferes with the IRS1/PI3K/AKT signaling pathway, contributing to IR, and ultimately participating in the pathological process of PCOS (Fig. 11). It was further confirmed that the crosstalk between RAS and insulin pathway was involved in the regulation of ovarian function and identified as a potential pathogenic mechanism of PCOS because intervention in this crosstalk significantly alleviated IR, and improved the reproductive and metabolic complications in PCOS mice.

In 1985, renin-like activity was identified in human ovarian follicular fluid, marking the beginning of research into the ovarian local RAS [41]. This discovery soon led to investigations into the classic pathway of the ACE-AngII-AT1 axis (AngII axis), exploring its role in several key physiological processes [4–9]. However, technological limitations and the complexity of ovarian function at that time prevented the formation of a clear consensus, causing this early wave of research to lose momentum. In the 21st century, new components of RAS were discovered, which together form the Ang(1-7) axis [42]. This discovery initiated the second wave of research. Currently, in other tissues, Ang(1-7) axis has been shown to mediate biological effects that counteract those of the AngII axis, and in short, balancing local RAS activity [43]. The third wave of research emerged during the COVID-19 pandemic. As we all know, ACE2, recognized as the key receptor of SARS-CoV-2 entry into human cells [44], has since become a major focus of research into local RAS across various tissues [45–47]. During this period, advanced technologies like scRNA-seq provided unprecedented new insights and details regarding local RAS.

Our results reveal that RAS components are widely expressed in ovarian cells, which have the conditions to form a local RAS independent of the circulatory system, and serve as one of the crucial systems to regulating the physiological process of the ovary. Previously, the independence of ovarian RAS was demonstrated in several aspects. Firstly, RAS components, including renin, AngII, Ang(1-7), and AT1/2 R, were discovered one after another in human ovaries and follicles [41, 48–51]. Subsequently, key evidence of this independence was provided by findings that the levels of renin and AngII in stimulated cycles and the preovulatory phase were significantly higher than those in circulation. Additionally, they were also found in the culture medium of human ovarian cells in vitro [52–55]. Furthermore, changes in ovarian RAS, rather than circulating RAS, were found to have a complex correlation with ovarian function [5, 56], though the underlying mechanisms remain unclear. AngII and Ang(1-7), bioactive polypeptides of RAS, are produced by renin cleaving AGT, with their proportions regulated by ACE and ACE2. These core components are widely expressed in various ovarian cell types, with significantly higher expression levels in stromal and epithelial cells. At the same time, notable aggregation of cells expressing RAS components was observed, particularly within the stromal and epithelial cells. Theca cells (stromal), granulosa cells, and luteal cells (epithelial) are key ovarian functional cells. Specifically, they are responsible for receiving signals from the hypothalamus-pituitary and directly transmitting them to oocytes [57, 58]. All the above findings provide more direct evidence for the role of RAS in regulating ovarian function and offer new clues and directions for further mechanistic exploration.

Ovarian RAS components fluctuate cyclically with the estrous cycle, which is controlled by gonadotropins (LH and FSH) released by the hypothalamus-pituitary. AGT, as the substrate of AngII and Ang(1-7), is highly expressed in all stromal (fibroblasts, mural and theca cells) and most epithelial (granulosa and luteal cells) cells. We found that AGT exhibits significant cyclical fluctuations, with the highest expression during estrus and the lowest during diestrus. In addition, ACE, which reflects AngII axis activity and is highly expressed in mural and granulosa cells, was also most expressed during estrus. Our findings, following a trajectory similar to that of gonadotropin, provide new evidence for RAS-mediated regulation of ovarian function by gonadotropins. As previously observed, the increase of intrafollicular AngII may facilitate oocyte maturation [59], ovulation [60], or follicular atresia [61], and corpus luteum regression [62]. On the contrary, renin, which is highly expressed in all stromal cells, exhibited its highest levels in diestrus. Given the role of renin in cleaving AGT to produce angiotensin I (AngI), it is speculated that this phase serves as preparation for substrate accumulation during the relatively long diestrus. Subsequently, the activation of the AngII and Ang(1-7) axes may be balanced by the expression levels of ACE and ACE2. In recent years, much research has focused on the novel Ang(1-7) axis. However, opposite to AngII, the level of Ang(1-7) in human follicular fluid was significantly lower compared to plasma (191 vs. 407 pg/mL) [51]. Although Ang(1-7) content is limited, the components of the Ang(1-7) axis have been gradually reported in the ovary [51], and the activation of the this axis appears to play a beneficial role [5].

The corpus luteum is essential for preparing the endometrium for embryo implantation and maintaining early pregnancy. The contribution of RAS in the corpus luteum to pregnancy has been uncovered through a long process of discovery. In short, it was found that prorenin (renin’s precursor) levels increased significantly in pregnant women, then dropped rapidly within 24 h after delivery, indicating that it originated from the mother rather than the fetus [63]. It was later confirmed that the rise in renin during pregnancy was dependent on ovarian function, with the ovary identified as the source [64]. A recent study further identified the corpus luteum as the key site and found a direct relationship between the number of corpora lutea and prorenin levels [65]. In addition, the role of placental RAS should not be overlooked. Indeed, chorionic prorenin levels are up to 1000 times higher than normal circulating prorenin levels [66], with maternal decidua being the major source of prorenin in the uteroplacental unit [67]. In summary, maternal circulating prorenin concentrations are associated with oocyte quality, early embryo development, embryo transfer, and implantation outcomes [68, 69]. Our results observed the contribution of luteal cells to AGT and renin production during the estrous cycle. Although it is regrettable that single-cell data of ovarian samples during pregnancy are unavailable, this remains a crucial and promising area for further research.

The multi-faceted crosstalk between insulin and local RAS signaling systems, observed in other organs, has also been demonstrated for the first time in the ovary. Interestingly, the relationship between RAS and the insulin pathway was initially discovered through the “side effects” of antihypertensive drugs. An epidemiological study showed that patients treated with ACE inhibitors or angiotensin receptor blockers had a lower risk of developing type 2 diabetes compared to those using other antihypertensive medications [70]. This prompted extensive research into their interaction mechanisms. It was found that AngII inhibits insulin receptor signaling in adipose cells [30], while increased activation of the Ang(1-7) axis can improve IR in adipose tissue [31]. Similar mechanisms have been observed in pancreatic RAS, where local RAS modulates glucose-stimulated insulin secretion in β cells [32, 33]. Moreover, this crosstalk has also been reported in cardiomyocytes, as well as in smooth and skeletal muscle cells [34, 71]. Given the widespread nature of this interaction [72], it is reasonable to infer that such crosstalk also exists in the ovary. We have verified this potential mechanism, which has not been reported previously.

The crosstalk between the local RAS and the IRS1/PI3K/AKT signaling pathway was preliminarily verified in PCOS mice with metabolic complications. When discussing ovarian IR, PCOS, a common reproductive, endocrine, and metabolic disorder, is often one of the first conditions that comes to our mind. Although not all women with PCOS are overweight, obese, or insulin resistant, a significant proportion are. A meta-analysis found that 61% (95% CI: 54-68%) of women with PCOS were either overweight or obese [73]. The recently updated PCOS guidelines also emphasize that IR is a key feature and recommend lifestyle interventions (like diet and/or exercise) or all women with PCOS [24]. As shown in our results and previous studies, PCOS is frequently associated with abnormal RAS activity in non-iatrogenic ovarian diseases, particularly in terms of total renin concentrations, ACE activity, and AngII levels [74–76]. Overall, These findings suggest excessive RAS activity in women with PCOS, especially in the AngII axis [7, 8]. From a molecular perspective, as indicated by our results, overactivation of the AngII axis affects IRS1/PI3K/AKT signaling pathway via AT1R. Detailedly speaking, although the direct effect of AT1R on IRS1 was not observed, as previously reported [70], the phosphorylation levels of PI3K and AKT were significantly restored. At the same time, some researchers have proposed additional crosstalk mechanisms involving other pathways such as JAK-2, JNK, ERK [77, 78], and dephosphorylation of the insulin receptor [79]. Moreover, the beneficial effects of Ang(1-7) on AngII-mediated IR further support this crosstalk mode from a reverse perspective [80].

While Candesartan showed a promising effect in treating PCOS mice in our research, it is important to note that Candesartan significantly improves systemic IR [81]. Similarly, other RAS blockade sites have shown benefits in improving IR and promoting weight loss [82, 83]. Therefore, it remains challenging to determine the specific role of Candesartan in the ovary independent of systemic effects. However, a population-based study of 23 million individuals confirmed that the use of RAS inhibitors was significantly associated with reduced risks of overall gynecologic cancers [84]. Furthermore, since AT1R is a G protein-coupled receptor involved in multiple downstream signaling pathways, further research is needed to identify the molecules interfering with insulin signaling. Finally, as the samples in our study were derived from mice, the observed phenomena need to be verified in humans, including the cyclical changes in human ovarian RAS and the therapeutic effect of Candesartan on women with PCOS.

In conclusion, this study revealed the dynamic changes of RAS during the estrous cycle at the cellular level and provided evidence that IR, caused by the crosstalk between AngII and IRS1/PI3K/AKT pathways, may be a potential underlying PCOS. This work utilized scRNA-seq technology to study ovarian RAS, providing new insights into this issue. Additionally, the study proposed a crosstalk mechanism that could improve the clinical symptoms of most women with PCOS who suffer from IR. Further clinical research is encouraged to clarify the physiological functions of ovarian RAS and confirm mechanism of the crosstalk mechanism identified in this study.

## Materials and methods

### Filtering and normalization of scRNA-seq data

The R (version 4.3.3) and RStudio (version 2023.12.1 + 402) were used for scRNA-seq data processing and analysis, and the study was based on the data structure and analysis method of the Seurat V4. Obtaining published scRNA-seq data (E-MTAB-11491, E-MTAB-12889) from ArrayExpress (https://www.ebi.ac.uk/biostudies/arrayexpress/). The Read10X read the scRNA-seq data, and the Harmony was used to integrate this data. To remove low quality cells, an adaptive filtering threshold approach was used based on high mitochondrial RNA content, extreme numbers of counts (count depth), and extreme numbers of genes per barcode. Cells were filtered based on the median absolute deviation from the median value of each metric across all cells. Specifically, the nFeature_RNA was set to express more than 300 genes and less than 7000 genes per cell, the nCount_RNA was set to UMI count greater than 1000 per cell and the largest top 3% cells were excluded, the mt_percent was set as the proportion of mitochondrial gene expression in total genes was less than 10% per cell, the HB_percent was set as the proportion of erythrocyte gene expression in total genes was less than 3% per cell. The NormalizeData was used for data normalization, and Cell cycle gene effect was regressed out using the CellCycleScoring function in the Seurat. The FindVariableFeatures was used to screen hypervariable genes. The FindNeighbors was used for data clustering. The UMAP was applied for dimensionality reduction and visualization.

### Cell type annotation of scRNA-seq data

First, clusters were assigned to known cell populations using cell type–specific markers obtained through the FindAllMarkers function. Multiple testing correction was performed using the Benjamini-Hochberg procedure. Second, the marker genes were determined by previous reports and CellMarker Database (Table S1), and the cell types could be determined according to the expression of marker genes in cell clusters. Finally, the determined cell names were marked on the corresponding cell clusters.

### Gene expression and protein-protein interaction analysis

The Idents was used to specify the comparison by cell type or estrus stage. The clusterProfiler was used for GO and KEGG analysis, and the gseKEGG was used for GSEA. Selecting gene set from the KEGG PATHWAY Database, including RAS [ko04614], Ovarian Steroidogenesis [ko04913], Progesterone-Mediated Oocyte Maturation [ko04914], Oocyte Meiosis [ko04114], and Insulin Signaling Pathway [Ko04910]. The protein-protein interaction network of the above gene set was obtained by STRING online analysis tool (https://string-db.org/), and the results were visualized by Cytoscape (version 3.10.2).

### Grouping design

The in vivo experiments on female C57BL/6 mice aged 6 weeks were divided into three groups: 24 mice were randomly divided into (a) Control group with 8 mice; (b) PCOS group with 8 mice and (c) MSCs group with 8 mice. (a) mice were fed a chow diet (10% fat) and injected with DHEA solvent (soybean oil) into the back of the neck for 21 days; (b-c) mice were fed a high-fat diet (60% fat) and injected with DHEA (6 mg/kg/day, dissolved in DMSO and fused with soybean oil) into the back of the neck for 21 days; (c) mice were also treated with Candesartan cilexetil (3 mg/kg/day, dissolved in DMSO and fused with sterilized water, MedChemExpress, USA) via intragastric administration for 21 days.

### Weight changes and vaginal smears

From the beginning to the end of modeling, the weight change of mice in each group was recorded every week. The vaginal smear was performed simultaneously during the last 8 days of modeling: Vaginal secretions of mice were taken out by dipping a cotton swab with normal saline and evenly spread on the glass slide. After natural air drying, the mice were fixed with absolute methanol for 15 min, stained with hematoxylin for 3 min, and slowly rinsed under running water for 5 min. After natural air drying, the mice were stained with eosin for 5 min and slowly rinsed under water for 3 min. Moreover, determine the period of estrus.

### Intraperitoneal glucose tolerance test

After completing 21 modeling days, mice in each group were fasted for 12 h, and initial blood glucose was measured. Glucose (2 g/kg) was administered by intraperitoneal injection, and blood glucose concentrations were measured from the tail vein of mice 15, 30, 45, 60, 90, 120, and 150 min after glucose administration using a glucometer.

### Serum hormone and biochemical test

Blood samples of mice were taken from postcava and centrifuged to get the serum. The ELISA kits (Jiangsu Meimian Industrial Co., Ltd., CN) were used to survey the levels of testosterone, anti-Müllerian hormone, luteinizing hormone, follicle-stimulating hormone, insulin, angiotensinogen, renin, angiotensin converting enzyme, and angiotensin II according to the instructions of manufacturers. The biochemical test were used to survey the levels of total cholesterol, triglyceride (TG), and low-density lipoprotein.

### Acquisition of mouse ovarian tissue

The animals were weighed on an electronic balance and the weights were recorded. After the mice were executed and placed on ice, the left and right ovarian tissues were rapidly dissected and removed. Connective tissues such as fat and fascia were removed on filter paper moistened with PBS buffer, then the tissues were rinsed 2–3 times with PBS and blotted dry. The weight of each ovarian tissue was measured on a microbalance and recorded.

### Hematoxylin-eosin staining

Mouse ovarian tissues were completely immersed in 4% polymerized formaldehyde solution and fixed for 24 h. After gradient alcohol dehydration and xylene transparent, the tissues are dipped in wax for 3–5 h and then paraffin embedded and sectioned. After dewaxing and rehydration, hematoxylin staining for 5–10 min, hydrochloric acid alcohol differentiation for 3–5 s, eosin staining for 2–3 min. After dehydration and sealing, the morphology of the tissue was visualized microscopically, and all levels of follicles were counted, including primordial, primary, secondary, antral/mature, and atretic follicles.

### qRT-PCR

The tissue RNA was extracted and reverse transcribed to cDNA. Messenger RNA (mRNA) abundance of *IRS-1, PI3K, AKT*, and *β-actin* in the ovary was determined using quantitative Real-time PCR (Thermo Fisher Scientific, USA). After Power Up SYBR Green Master Mix premix (Thermo Fisher Scientific, USA), forward primer, reverse primer (Table 1) and ddH_2_O were added, add 9 μl of reaction solution and 1 μl of sample cDNA to each well of the 96-well plate, and do 3 replicates for each sample. The program was run, the data was exported when it was finished, and a record of the experiment was kept.

**Table 1:** Sequences of the primers used in qRT-PCR.

TARGET GENE	PRIMER	NUCLEOTIDE SEQUENCE
*Irs-1*	*F*	ACCAGCCCTTAGGCAGCAAT
	*R*	GAAGCTGATGCTGGCATAGTTG
*Pi3k*	*F*	GGGCAGTTAAGAAGCACAATG
	*R*	GCAGGAGAGTCTTTCCAATG
*Akt*	*F*	GATCATGCAGCACCGGTTCT
	*R*	GGCGTGATGGTGATCATCTG
*β-actin*	*F*	AGCCATGTACGTAGCCATCC
	*R*	TCCCTCTCAGCTGTGGTGGTGAA

### Western blot

Protein lysate was prepared by mixing RIPA with PMSF (100 mM) at a ratio of 100:1. Then, 20 μl of the protein lysate was added to 1 mg of tissue for grinding. The lysate was kept on ice for 30 min and centrifuged at 15,000 rpm for 10–15 min at 4 °C. The supernatant was collected. SDS loading buffer was added and the system was metal bathed at 100 °C for 15–20 min. The gel was dispensed and the electrophoresis tank was assembled with the prepared protein samples. Electrophoresis was performed at 80 V for 30–50 min, followed by a change to 100–120 V for 2 h. The wet transfer system was then assembled and electrified at 200 mA at a constant current for 2 h. The membrane was blocked in a solution of 5–10% skimmed milk on a shaking bed for 1–2 h at ambient temperature, followed by incubation with the primary antibody at ambient temperature for 12 h with shaking. The primary antibodies were diluted (IRS-1, 1:1000, ABclonal, CN; PI3K,1:5000, Proteintech, USA; Phospho-PI3K, 1:2000, Affinity, USA; AKT, 1:2000, Proteintech, USA; Phospho-AKT, 1:2000, Affinity, USA; β-actin, 1:6000; Abcam, UK) and used. Depending on the properties of the primary antibody, the membrane was then incubated with the corresponding secondary antibody for 1 h at ambient temperature with shaking. Protein bands on the membrane were visualized using an ECL luminescent agent in a dark room.

### Quantification and statistical analysis

The organ coefficient of ovaries = ovarian weight (mg) / body weight (g). HOMA-IR = FBG × FINS/22.5; HOMA-IS = 1/HOMA-IR; HOMA-β = 20 × FINS/(FBG-3.5). The formula for analyzing the qRT-PCR results of mRNA transcription levels can be referred to as follows: 2^−△△ct^ = 2^− [(Average ct value of the target gene in the test group) − (Average ct value of the reference gene in the test group)] − [(Average ct value of the target gene in the control group) − (Average ct value of the reference gene in the control group)].^ The Western blot results for protein translation levels were analyzed using Image J software to measure the grayscale values. To ensure the accuracy and credibility of the experiment, we conducted at least 3 biological replicates in each group, assigning at least 3 mice to ensure the reliability and robustness of the experiment. The Shapiro-Wilk test was used to test whether the data conforms to the normal distribution. All data were shown as the mean ± SEM. The F-test (joint hypotheses test) is used to test whether the variance between each group of data is equal. GraphPad Prism 10.0 was used for statistical analysis of results from the two groups through t-test, and the more than two groups through two-way ANOVA (scRNA-seq results were compared in pairwise, and laboratory results were compared with PCOS group), and significance was taken as *P* < 0.05.

## Supplementary information

Supplementary Table 1

Original Data-Western Blot Gels

## Data Availability

The datasets used and/or analyzed during the current study, as well as the code, are available from the corresponding author upon reasonable request.
