# Supplementary material for: Single cell RNA sequencing reveals the role of local renin-angiotensin system in regulating ovarian physiological cycle and promoting PCOS
Source: Cell Death Discov. 2025 May 27;11:255. doi: 10.1038/s41420-025-02531-8 (PMC12116893; doi:10.1038/s41420-025-02531-8)

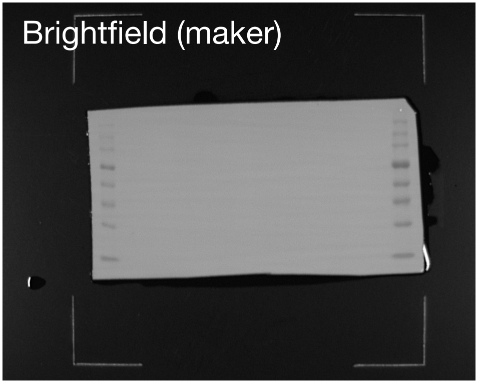

Developed by ECL

Name: β-actin

Host: Rabbit

Producer: abcam

Catalog: ab227387

Application: 1:6000

Clonality: Polyclonal

Observed MW: 42kDa

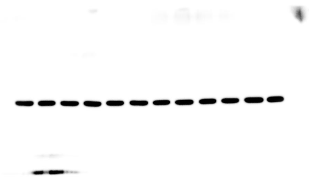

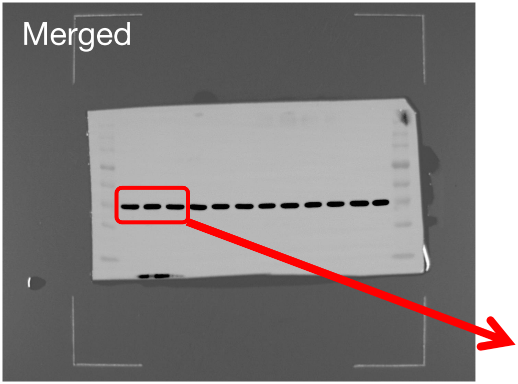

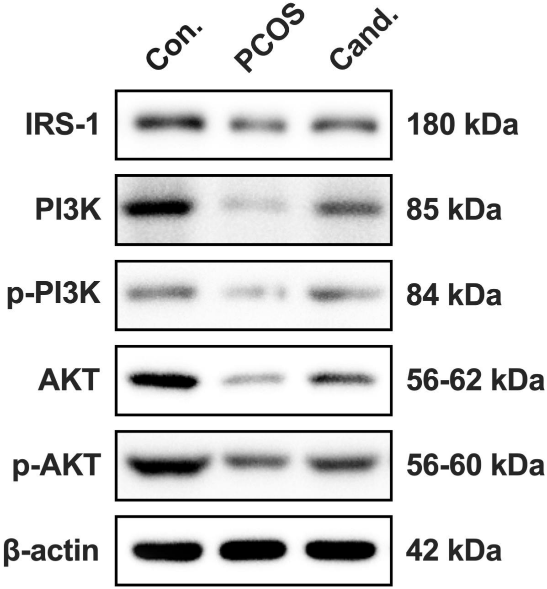

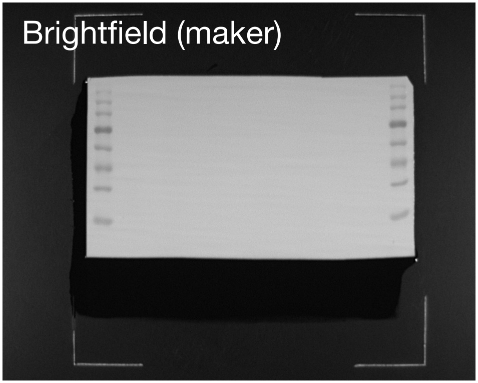

Developed by ECL

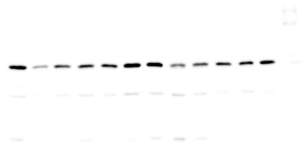

Name: AKT

Host: Rabbit

Producer: Proteintech

Catalog: 10176-2-AP

Application: 1:2000

Clonality: Polyclonal

Observed MW: 56-62kDa

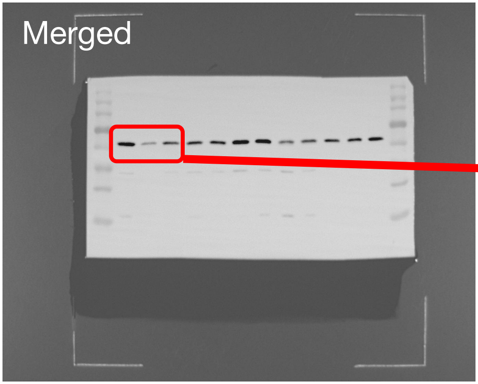

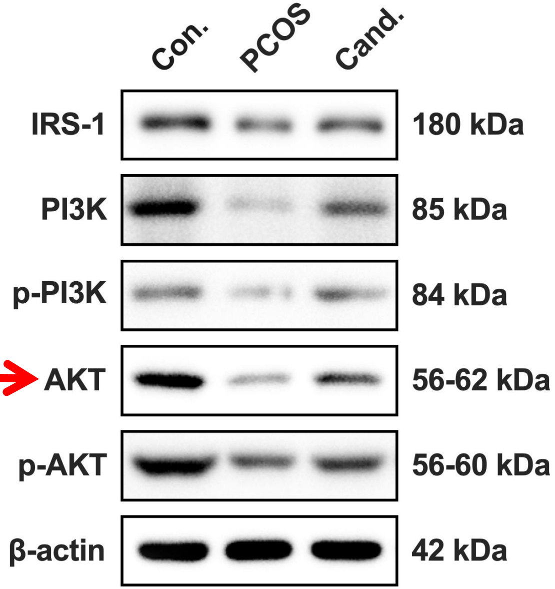

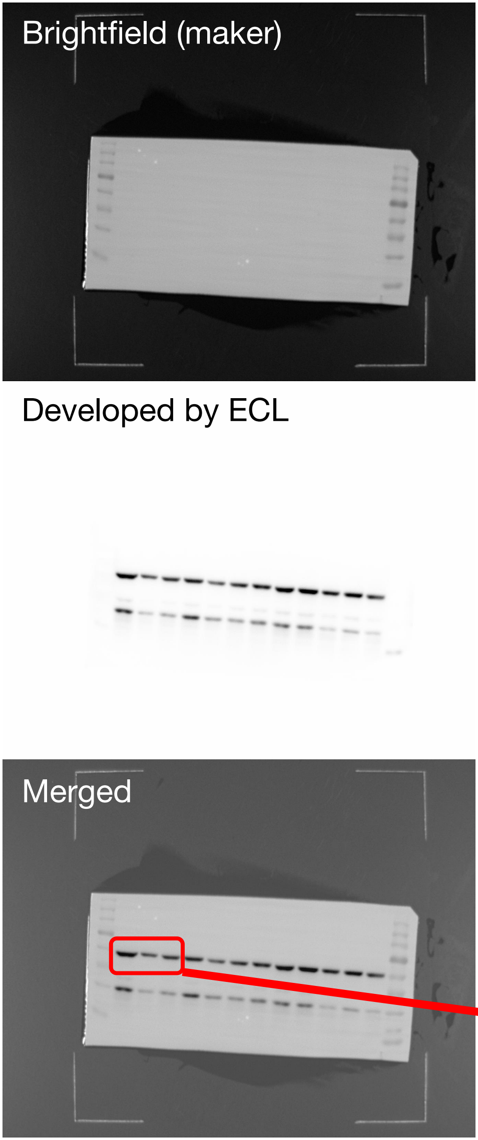

Name: Phospho-AKT

Host: Rabbit

Producer: Affinity

Catalog: AF0016

Application: 1:2000

Clonality: Polyclonal

Observed MW: 56-60kDa

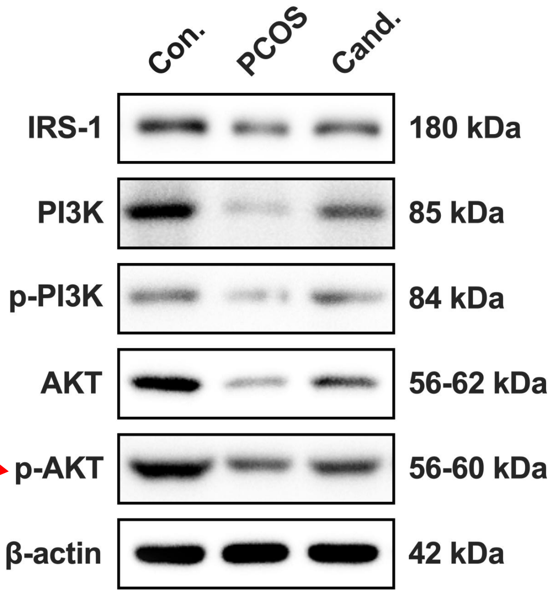

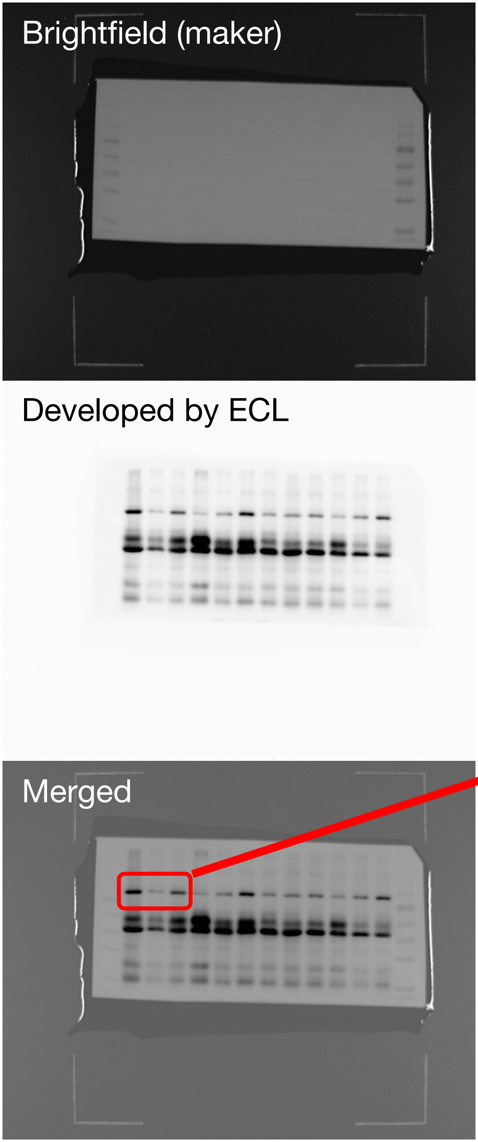

Name: PI3K p85

Host: Mouse

Producer: Proteintech

Catalog: 60225-1-Ig

Application: 1:5000

Clonality: Monoclonal

Observed MW: 85kDa

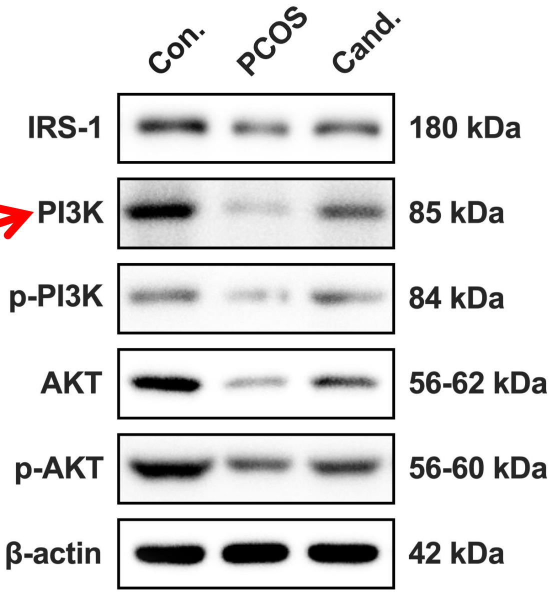

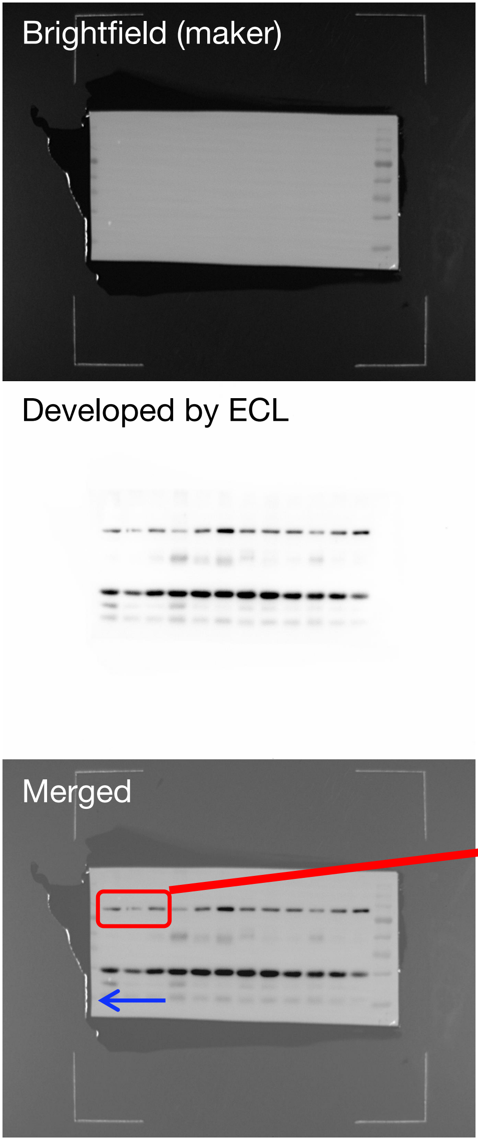

Name: Phospho-PI3K p85

Host: Rabbit

Producer: Affinity

Catalog: AF3242

Application: 1:2000

Clonality: Polyclonal

Observed MW: 84kDa

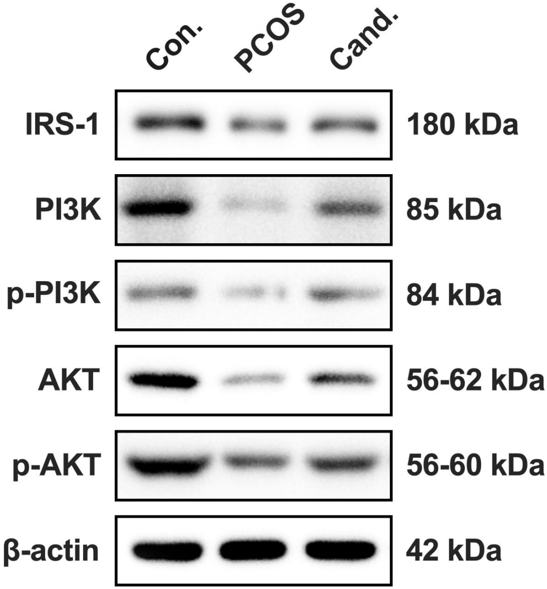

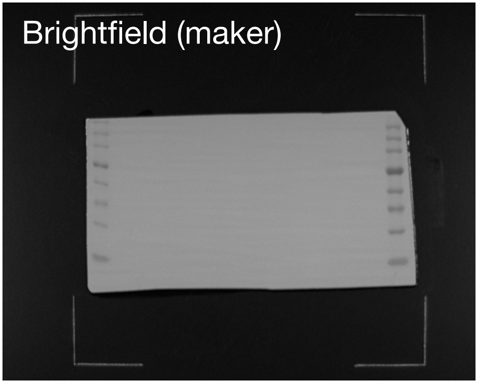

Developed by ECL

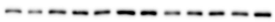

Name: IRS1

Host: Rabbit

Producer: ABclonal

Catalog: A16902

Application: 1:1000

Clonality: Polyclonal

Observed MW: 180kDa

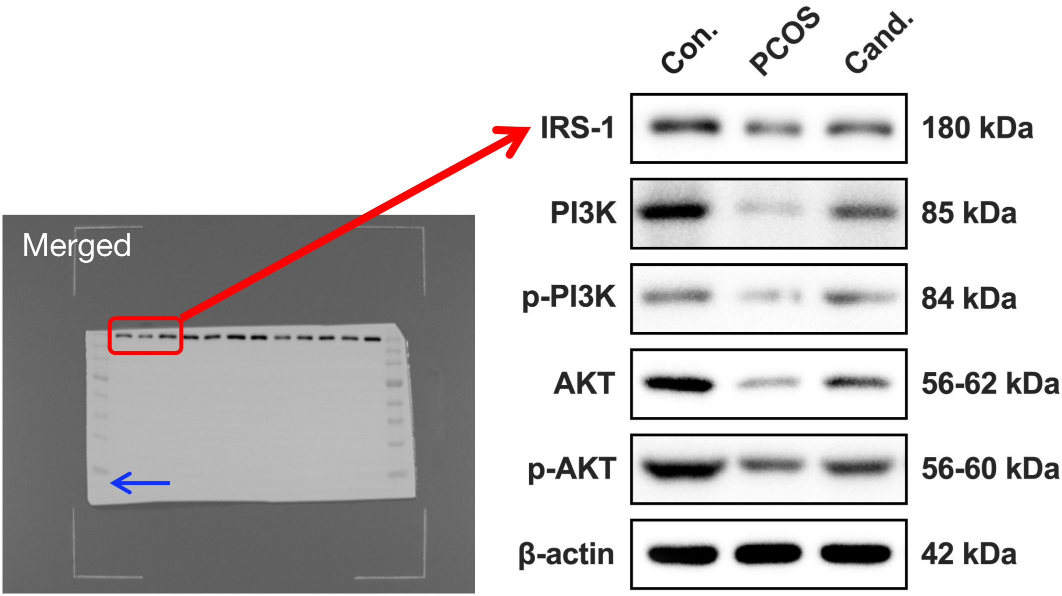

Supplement: Supplementary file 2 — Original Data-Western Blot Gels [file 41420_2025_2531_MOESM2_ESM.pdf]
